# MicroRNA-302 switch to identify and eliminate undifferentiated human pluripotent stem cells

**DOI:** 10.1038/srep32532

**Published:** 2016-09-09

**Authors:** Callum J. C. Parr, Shota Katayama, Kenji Miki, Yi Kuang, Yoshinori Yoshida, Asuka Morizane, Jun Takahashi, Shinya Yamanaka, Hirohide Saito

**Affiliations:** 1 Department of Life Science Frontiers, Center for iPS Cell Research and Application (CiRA), Kyoto University, Kyoto, Japan; 2 Department of Clinical Application, Center for iPS Cell Research and Application (CiRA), Kyoto University, Kyoto, Japan; 3 Gladstone Institute of Cardiovascular Disease, San Francisco, CA 94158, USA

## Abstract

The efficiency of pluripotent stem cell differentiation is highly variable, often resulting in heterogeneous populations that contain undifferentiated cells. Here we developed a sensitive, target-specific, and general method for removing undesired cells before transplantation. MicroRNA-302a-5p (miR-302a) is highly and specifically expressed in human pluripotent stem cells and gradually decreases to basal levels during differentiation. We synthesized a new RNA tool, miR-switch, as a live-cell reporter mRNA for miR-302a activity that can specifically detect human induced pluripotent stem cells (hiPSCs) down to a spiked level of 0.05% of hiPSCs in a heterogeneous population and can prevent teratoma formation in an *in vivo* tumorigenicity assay. Automated and selective hiPSC-elimination was achieved by controlling puromycin resistance using the miR-302a switch. Our system uniquely provides sensitive detection of pluripotent stem cells and partially differentiated cells. In addition to its ability to eliminate undifferentiated cells, miR-302a switch also holds great potential in investigating the dynamics of differentiation and/or reprograming of live-cells based on intracellular information.

Induced pluripotent stem cell (iPSC) technology holds great promise for regenerative medicine while circumventing the ethical and practical issues surrounding the use of stem cells from embryonic sources. Furthermore iPSC technology allows for personalized medicine that give targeted therapy without immune complication. In addition, iPSC technology is proving to be a vital tool for disease modelling, creating more realistic cell-models from patients with all the complicated genetic and epigenetics pre-programmed. Since the initial discovery of the induced reprogramming mechanism for mouse and then human cells in 2006 and 2007 respectively, iPSCs have been differentiated into to numerous types of somatic cells^1^,^2^. Methods for cell reprogramming follow broadly two main strategies: (1) Direct cell-fate conversion in which genetic manipulation is required to overexpress transcription factors and/or microRNAs. (2) The use of compounds, cytokines and/or recombinant signal peptides that stimulates reprogramming. The latter method is preferred for clinical application but often gives lower efficiencies. These protocols have largely been adapted from the pre-existing methods using embryonic stem cells^3^,^4^,^5^. However, in the case of iPSCs, studies suggest the differentiation is highly dependent on the line, which may cause some practical issues for therapy^6^,^7^.

An important issue to be solved before iPSC-base therapies enter the clinic is the carryover of undifferentiated iPSCs, partially differentiated cells, and wrongly differentiated cell types during transplantation. This problem arises, as no protocol is 100% efficient in generating the correct lineage let alone the target cell type. Furthermore, the differentiation efficiency can vary greatly depending on which iPSC clone is used because of the variable expression of key genes, including ones driven by human endogenous retrovirus type-H long-terminal repeats, which may be inhibitory to certain lineages^8^,^9^. In one study, several iPSC lines differentiated into midbrain neuronal lineage were found to be differentiation-defective, and the resulting cell population contained residual iPS cells that caused graft overgrowth when transplanted to mice. Even when no residual iPS cells were detected, the transplanted cells from certain lines lead to graft overgrowth due to partially differentiated cells^8^. Therefore, there is a real need to not only make sure transplanted cells are devoid of residual pluripotent cells but also partially differentiated cells that may lead to graft overgrowth. Recent tumorigenesis experiments have found as few as 100 pluripotent stem cells transplanted to Severe Combined Immunodeficiency (SCID) mice can lead to teratoma growth^10^,^11^.

For certain cell types, there are no effective cell-surface or intracellular markers for their positive selection by cell sorting. Furthermore, in some cases, a mix-culture of cells, that excludes harmful cells to cause teratoma formation or graft overgrowth, is required. In the above cases, ideally we would use a general tool that can remove the undifferentiated or partially differentiated cells, while also being applicable to any differentiation protocol (Fig. 1a, top). Here we have established such a method, which can selectively identify undifferentiated and partially differentiated cells with high-resolution. The method is simple and cost-effectively, and can also be easily scaled up to handle millions of cells. It is noteworthy that our method is the only one capable of interrogating the intracellular information of living cells. Comparatively, most existing technologies are restricted to information displayed on the cell surface.

Our technology, microRNA switches (miR-switches), were encoded on modified mRNA (modRNA)^12^,^13^ that post-transcriptionally regulates fluorescent reporters in response to the activity of the human miRNA-302/367 cluster expressed in living cells (Fig. 1a, bottom). This cluster is important for maintaining the self-renewal of stem cells and particularly in the primed state of pluripotency^14^,^15^,^16^,^17^. The high dynamic range in expression between pluripotent and differentiated cells of miR-302a allows for very sensitive detection. In the present work, we could reproducibly detect spiked hiPSCs down to 0.05% of the total cell number using the miR-302a switch. Furthermore, due to the dynamic regulation of the fluorescence we could identify cells transitioning from the undifferentiated to differentiated stage at high-resolution, thus allowing us to discriminate sub-populations, such as partially differentiated cells, and isolate or eliminate them. In addition, by sorting hiPSC spiked midbrain dopaminergic (mDA)-like neuronal cell cultures with miR-302a switch, we could prevent teratoma formation in a standard *in vivo* tumorigenicity assay on mice. Lastly, we placed the miR-302a switch in the control of puromycin selection to automatically and selectively remove contaminating hiPSCs, thus removing the necessity for cell sorting, which is time consuming and can damage the cells.

## Results

### Generation of live-cell reporters to detect pluripotency-specific miR-302/367 activity

**Figure 1:**
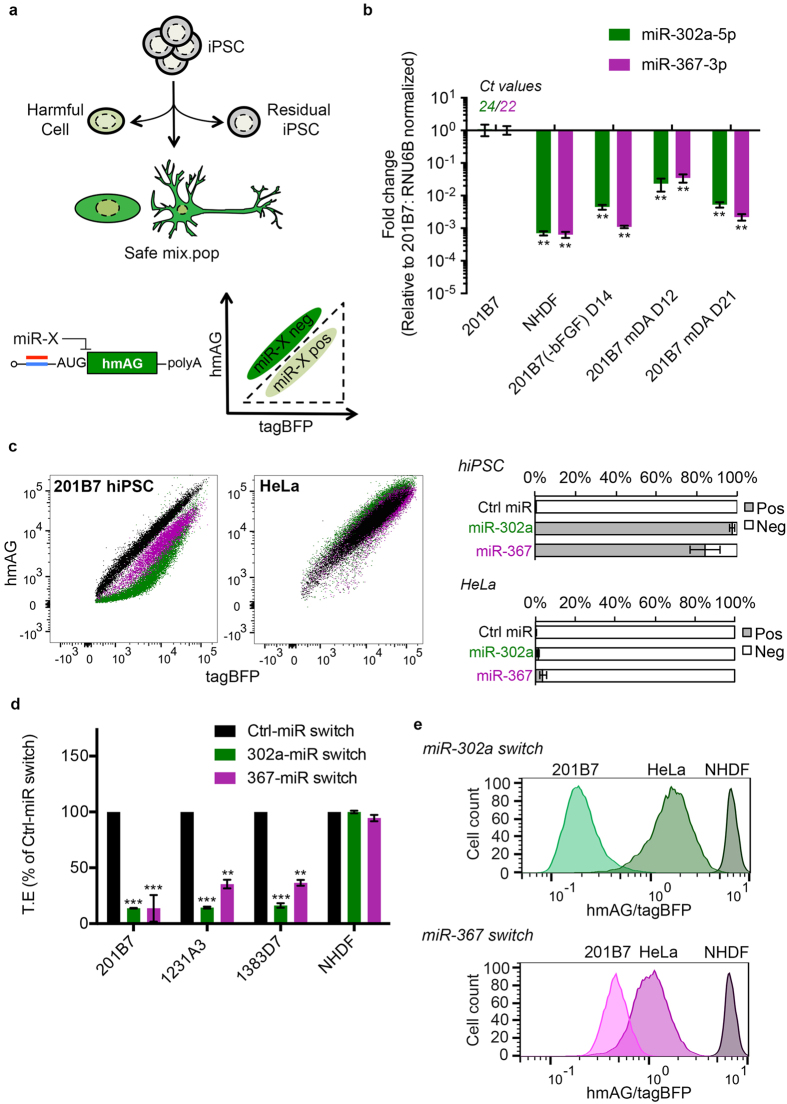
miR-302a and 367 switches specifically detect hiPSC cells. (**a**) miR-302a switch can remove undifferentiated or partially differentiated cells before transplantation. miRNA binding to the 5′UTR of the hmAG reporter causes translation repression. The dotted outline on the dot-plot corresponds to the miR-pos fraction. (**b**) hsa-miR-302a-5p and -367-3p are specifically expressed in 201B7 hiPSCs relative to NHDF and downregulated in spontaneously differentiated 201B7 cells and 201B7-derived mDA cells (n = 3 for all groups). (**c**) Representative dot plots of 201B7 and HeLa transfected with either 45 ng of Ctrl- (black dots), miR-302a (green) or miR-367 (purple) switches mRNA and 90 ng of tagBFP internal control. Right panel shows the percentage of 302-pos and 302-neg cells (n = 3 for all groups). (**d**) Percentage of translation efficiency (T.E., geometric mean of hmAG/geometric mean of tagBFP) of three hiPSC lines and NHDF cells transfected with Ctrl- (black), miR-302a (green) or miR-367 (purple) switches (n = 3 for all groups). (**e**) Representative histograms of the translation efficiency of the raw fluorescence signal (hmAG/tagBFP) for 201B7, HeLa and NHDF transfected with either miR-302a (top) or miR-367 (bottom). Error bars represent the SEM of three independent experiments.

We focused on the miR-302/367 cluster as previous reports have illustrated this cluster to be intrinsically involved in the regulation of pluripotency^15^,^17^,^18^,^19^. We first confirmed the specific and high level of expression of both hsa-miR-302a-5p (herein referred to as miR-302a) and hsa-miR-367-3p (herein referred to as miR-367) in three feeder-free human iPSC lines (201B7, 1231A3, and 1383D7). We found the expression of miR-302/367 in the iPSC lines to be 10^2^,^3^,^4^,^5^,^6^-fold greater than spontaneously differentiated 201B7 cells (cells cultured without basic growth factor (bFGF) for 2 weeks), 201B7-derived mDA cells, standard culture lines of NHDF and HeLa, and lastly primary hepatocytes and renal cells (Fig. 1b and Supplementary Fig. 1a).

**Figure 2:**
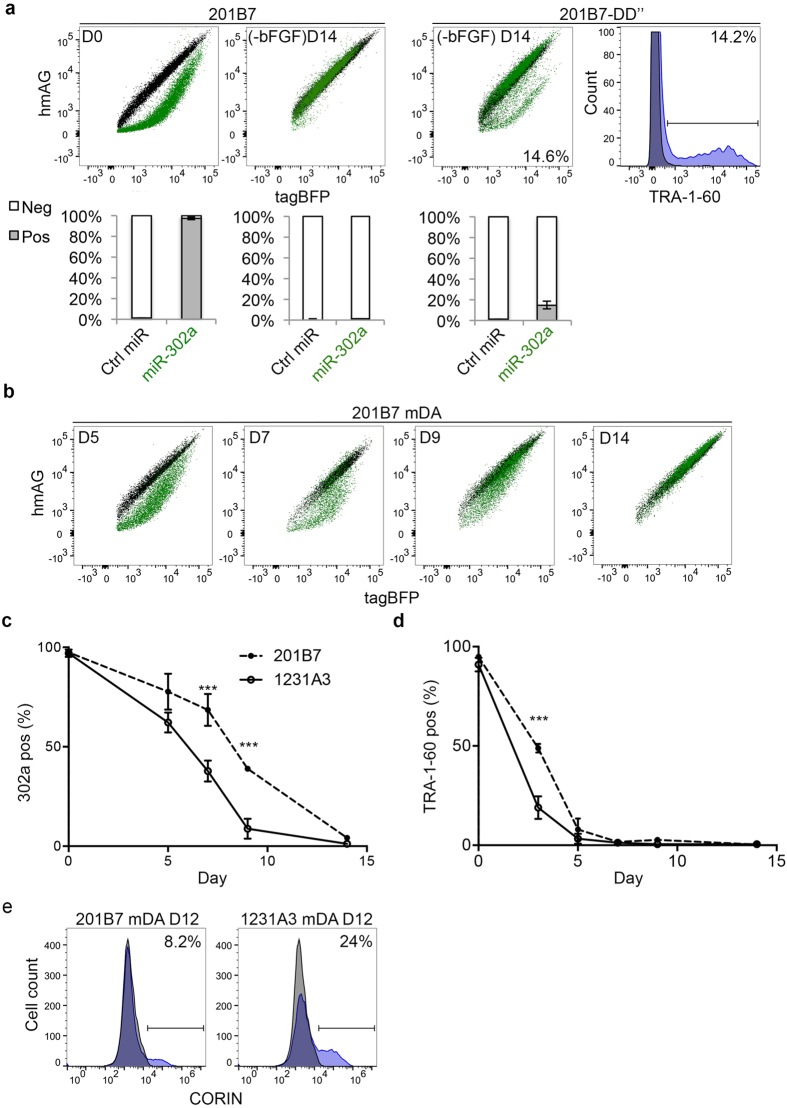
miR-302a switch distinguishes hiPSCs and hiPSC-derived differentiated cells. (**a**) LEFT PANELS Representative dot plots of 201B7 cells transfected with either 45 ng of Ctrl-miR (black) or miR-302a (green) switches and 90 ng of tagBFP at day 0 or day 14 in hiPSC medium (-bFGF) condition. *Below* Percentage of 302-pos and 302-neg cells (n = 3 for all groups). RIGHT PANELS Representative dot plot of the 201B7-DD″ after 14 days differentiation transfected with either Ctrl-miR (black) or miR-302a (green) switches. The far right plot shows the same cells stained with Alexa 647-conjugated IgG control (black shade) or Alexa-647-conjugated anti-TRA-1-60 antibody (blue shade). *Below* Percentage of 302-pos and 302-neg cells (n = 3 for all groups). (**b**) Representative dot plots of 201B7 cells differentiated with the mDA protocol and transfected with 45 ng of miR-302a switch (green) and 90 ng of tagBFP. Cells were analysed at days 5, 7, 9 and 14 of the differentiation. (**c**) Percentage of 302-pos cells versus time (day 0 to 14) for cells derived from 201B7 or 1231A3 hiPSC lines (n = 3 for all groups). (**d**) Percentage of TRA-1-60-pos cells versus time (day 0 to 14) for cells derived from 201B7 or 1231A3 lines (n = 3 for all groups). (**e**) Representative histograms of Alexa 488-conjugated IgG staining (black shade) or CORIN staining (blue shade) for mDA cells derived from 201B7 or 1231A3 at day 12. Error bars represent the SEM of three independent experiments.

**Figure 3:**
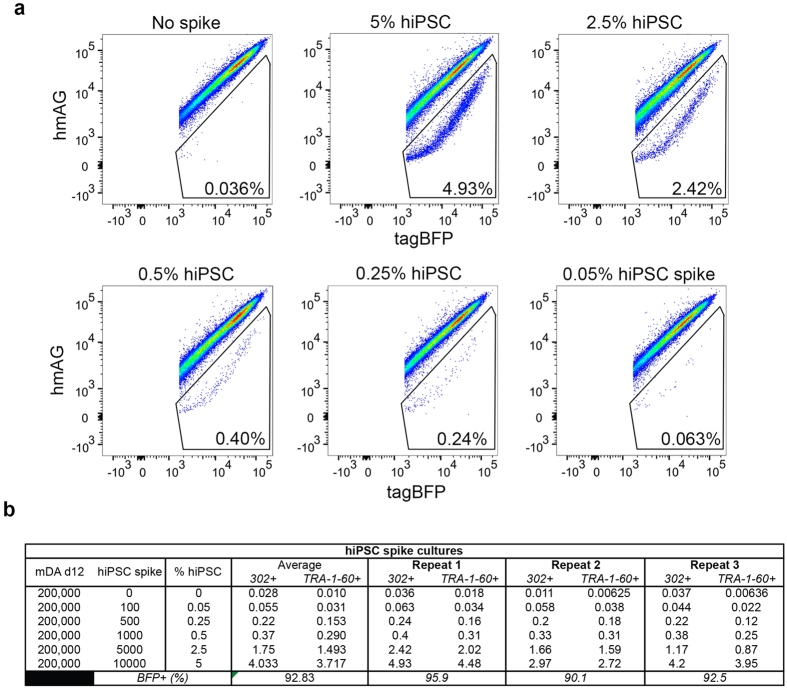
Detection sensitivity for spiked hiPSC cells with miR-302a switch. (**a**) Representative dot plots of hmAG vs. tagBFP fluorescence for day 12 mDA cells spiked with 5%, 2.5%, 0.5%, 0.25% and 0.05% hiPS cells and transfected with 90 ng of miR-302a switch mRNA and 180 ng of tagBFP, and stained with anti-TRA-1-60 antibody before flow cytometry (Supplementary Fig. 4). (**b**) The table summarises percentage of 302-pos, 302-neg, TRA-1-60-pos, and TRA-1-60-neg cells for different hiPSC spike numbers (three independent experiments each).

We generated *in vitro* transcribed modRNA that encoded miR switches that can sensitively and dynamically respond to the activities of miR-302a and miR-367 in living cells. Translational repression of the reporter protein (human codon optimised azamin green, hmAG) occurs when the mature miRNA binds to an antisense sequence that is optimally placed within the 5′-UTR (Fig. 1a, bottom) of the mRNA^12^,^13^. Normalizing this reporter expression to an internal fluorescent control (tag blue fluorescent protein, tagBFP) that is co-transfected produces normalized translation efficiency (T.E, refer to methods). In the absence of the miRNA, the hmAG:tagBFP expression ratio is approximately 1:1^13^. However with the presence of the miRNA, translation from the reporter is repressed, causing a downward shift in hmAG fluorescence on the dot-plot as illustrated in Fig. 1a, with cells within dotted-gate being the positive (pos) fraction responding to the miR-switch. 201B7 cells transfected with either miR-302a (green, Fig. 1c) or miR-367 (purple, Fig. 1c) switches are spatially resolved from cells transfected with miR switch containing no miRNA antisense sequence (Ctrl-miR switch, black dots), whereas most HeLa cells transfected with the three switches overlapped each other (Fig. 1c). From these results, we decided to focus on miR-302a switch for the following reasons: (1) From three independent experiments, the percentage of hiPS cells responsive to the miR-302a switch (positive = 97.05%, SEM ± 3.18) than those that responded to the miR-367 switch (pos = 81.45%, SEM ± 19.97) (Fig. 1c); (2) When measuring the translational efficiency from the dot plots the miR-302a switched-transfected hiPS cells gave a higher fold-change than miR-367 switch-transfected cells (Fig. 1d); (3) And the miR-302a switch could completely separate 201B7 from NHDF and HeLa cells, whereas the miR-367 switch did not (Fig. 1e).

We next checked whether the repression on translation efficiency by the miR-302a switch was indeed specific to the miR-302a-5p activity. We co-transfected 201B7 cells with 15 pmol of either a control (green bar) or miR-302a specific (grey bar) miRNA inhibitor (Supplementary Fig. 1b). Co-transfection of the 302a miRNA inhibitor allowed for recovery of the T.E. (Supplementary Fig. 1b,c). We next co-transfected varied amounts of the miRNA inhibitor in 201B7 and a miRNA mimic in HeLa cells to simulate the dynamic range of the miR-302a switch. In transfected cells, the increasing amount of 302a miRNA inhibitor caused a gradual recovery in the translational efficiency (hmAG/tagBFP). In contrast, the increasing amount of the 302a mimic caused a gradual repression in the translational efficiency in HeLa cells (Supplementary Fig. 1d). These results indicate miR-302a activity regulates the miR-302a switch specifically.

### miR-302a switch effectively separates hiPSCs and hiPSC-derived differentiated cell**s**

In addition to checking standard culture lines and primary cells, we also tested our miR-302a switch on hiPSC-derived differentiated cells. We spontaneously differentiated hiPSCs by removing bFGF from the medium for 14 days. Again, proving that miR-302a switch is specific to pluripotent stem cells, there was a complete overlap of 201B7-derived spontaneously differentiated cells transfected with either Ctrl-miR or miR-302a switch (Fig. 2a), suggesting that miR-302a switch distinguishes hiPSCs and differentiated cells. Furthermore, when using the same differentiation protocol on a differentiation-defective version of 201B7, 201B7-DD″ (see methods and Supplementary Fig. 2), after 14 days we could detect residual differentiation resistant cells (14.6 ± 3.4%) when transfecting miR-302a switch (Fig. 2a). This ratio was very similar to the percentage of cells that were positive for TRA-1-60 (a cell surface marker for hPSCs) antibody (14.2 ± 2.5%).

### Monitoring differentiation dynamics and efficiency using miR-302a switch

We next tested our miR-302a switch on another hiPSC-derived differentiated cell type. We produced mDA neuronal cells using an established protocol (Supplementary Fig. 3a,b)^5^,^8^,^20^. After 14 days, like the results for spontaneously differentiated cells, the mDA neurons transfected with miR-302a switch overlapped with those transfected with Ctrl-miR switch (Fig. 2b, D14). These results further confirm that miR-302a switch can reproducibly distinguish hiPSCs and their differentiated cells. Moreover, the gradual recovery in translational efficiency in the representative dot plots from days 5, 7 and 9 (Fig. 2b), indicates that the level of active miR-302a in the cells gradually decreased during differentiation. Using two hiPSC lines, we analysed the percentage of 302-positive (302-pos) cells at the same time points. Interestingly, we saw significant differences between 201B7 and 1231A3 lines. This difference was most noticeable at days 7 and 9, when the 302-pos cell population in 201B7 differentiated cells was 68.55 ± 7.97% and 38.95 ± 0.15%, respectively, but in 1231A3 it was lower at 37.78 ± 5.23% and 8.81 ± 4.99% (Fig. 2c). In both lines, the percentage of TRA-1-60-pos cells decreased to near background level by day 5, suggesting that TRA-1-60 antibody cannot distinguish these lines after 5 days of differentiation (Fig. 2d). To assess the differentiation efficiency to the mDA identity, we stained the cells for CORIN expression, since staining for this cell-surface protein was recently found to enrich mDA cells^20^, and analysed the cells by flow-cytomery at day 12. The 1213A3 line consistently produced a higher number of CORIN-pos cells than the 201B7 line (see representative histograms in Fig. 2e). These data suggests miR-302a switch can detect partially differentiated cells (*e.g.*, the cells after 9 days of differentiation in Fig. 2b,c). Thus, miR-302a switch can assay the differentiation dynamics and optimal condition and clone for directed differentiation.

### Sensitive detection of spiked-hiPSCs using miR-302a switch

To facilitate the therapeutic application of iPSC technology, it is important to detect and eliminate residual harmful cells with high sensitivity. Thus, as a next step, we moved from single- to co-cultured cell populations to check whether miR-302a switch could isolate hiPSCs from differentiated cells in a mixed culture. To this end, we took 201B7-derived mDA day 12 cells (2 × 10^5^ cells) and spiked with a decreasing number of hiPSCs, from 1 × 10^4^ (5% spike) to just 100 cells (0.05% spike) (Fig. 3). The entire volume of the cell-suspension was analysed by flow-cytometry. We successfully detected hiPSCs at all the spiked levels with miR-302a switch. The percentage of 302-pos cells closely matched that of TRA-1-60-pos cells (Fig. 3b and Supplementary Fig. 4).

### miR-302a switch sorting on partially differentiated mDA neuronal cultures

**Figure 4:**
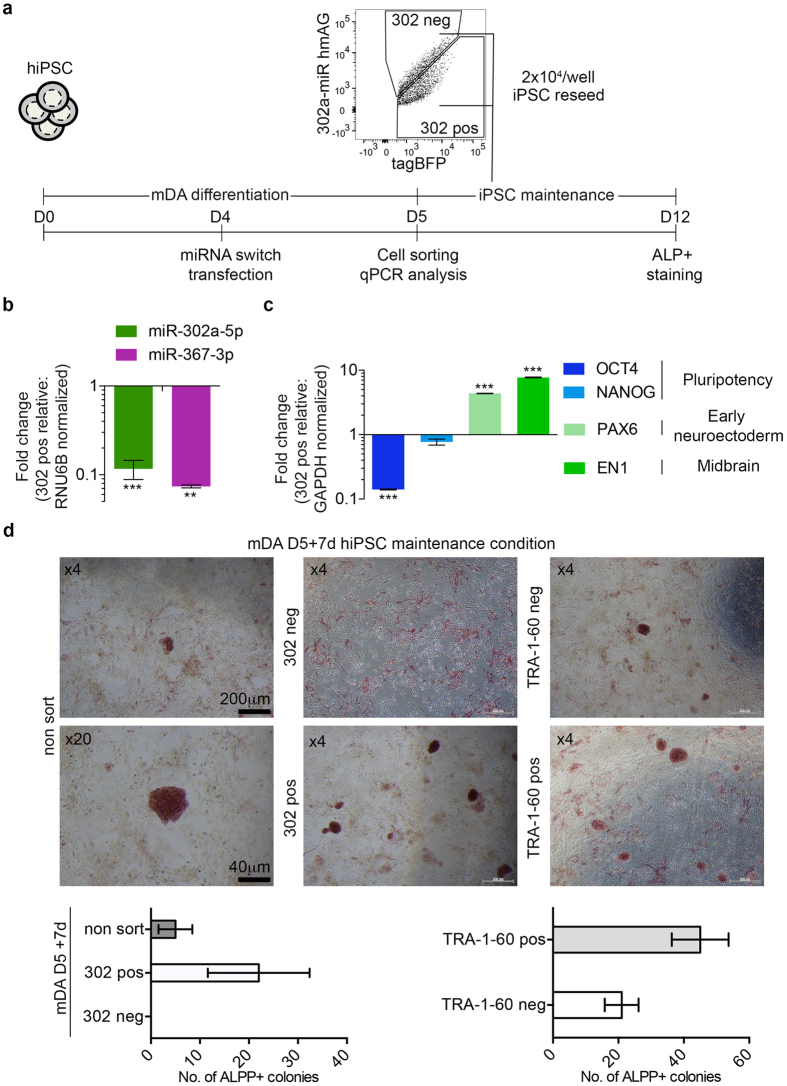
Sorting of partially differentiated mDA cells using the miR-302a switch. (**a**) Schematic of the experimental procedure. hiPSCs were partially differentiated through the mDA differentiation protocol for 4 days and passaged to a new 6-well plate for reverse transfection of miR-302a switch and sorted into 302-pos and 302-neg fractions for RT-qPCR analysis or recultured for later alkaline phosphatase staining. (**b**) Fold change in miR-302a-5p and 367-3p expression for the 302-neg fraction relative to the 302-pos fraction and normalized with RNU6B expression from snap-frozen cells sorted on day 5 (n = 3 for all groups). (**c**) Relative RT-qPCR gene expressions from 302-neg to 302-pos fractions and normalized by GADPH from snap-frozen cells sorted on day 5 (n = 3 for all groups). (**d**) Representative bright-field images of sorted cells recultured in hiPSC medium for a further 7 days and (below) the counted number of ALP-pos colonies. The TRA-1-60-pos and -neg fractions were sorted from non-transfected cells stained with an anti-TRA-1-60 antibody. Error bars represent the SEM of three independent experiments.

As a proof of principle that our miR-302a switch can be used to make cultures safer for transplantation, we deliberately suspended the mDA differentiation of hiPSC cells at day 4 (cells after 4 days of differentiation contain undifferentiated and/or partially differentiated cells in our mDA induction protocol). These cells were then transfected with miR-302a switch and underwent FACS the following day to separate 302-pos and 302-negative (302-neg) cells (see scheme in Fig. 4a). We then re-cultured an equal number of 302-pos and 302-neg sorted cells to new plates and grew them for a further 7 days in hiPSC-culture medium to promote the growth of any residual harmful cells. We first confirmed the 302-pos and 302-neg sorted fractions from cells on day 5 for RNA analysis. We measured the expression of miR-302a and miR-367 and then expression of key pluripotent-, neuroectoderm- and mDA-associated mRNAs. As expected, the 302-neg sorted fraction contained a reduced level of both miR-302a and miR-367 relative to 302-pos sorted fraction (Fig. 4b). Furthermore, the 302-neg sorted fraction had a lower expression level of the pluripotency marker *OCT4*, higher expression levels of the neuroectoderm marker *PAX6* and of the midbrain marker *EN1* (Fig. 4c). After 7 days of continuous culture in hiPSC culture medium, the cells were fixed and stained with an alkaline phosphatase (ALP) kit to detect hiPSC colony formation. This staining is often used to screen for colonies during iPSC reprogramming^21^. Over three independent sorting experiments, a significant number of ALP+ iPS-like colonies were observed in wells seeded with either non-sorted (5 ± 3 colonies) or 302-pos sorted cells (22 ± 6) (Fig. 4d). In contrast, we saw no colonies in the wells re-cultured with 302-neg sorted cells. For comparison, we also sorted day 5 mDA cells based on TRA-1-60 staining and as above re-cultured for 7 days an equal number of the TRA-1-60-pos and TRA-1-60-neg cells. As expected, wells seeded with TRA-1-60-pos cells had an even higher number of colonies (45 ± 5 colonies). Interestingly, TRA-1-60-neg cells also went on to form ALP^+^ colonies (21 ± 3 colonies), illustrating the high sensitivity of miR-302a switch, which can be used to remove harmful overgrowing cells.

### miR-302a switch-sorted hiPSC-spiked co-cultures could prevent teratoma formation in SCID mice

**Figure 5:**
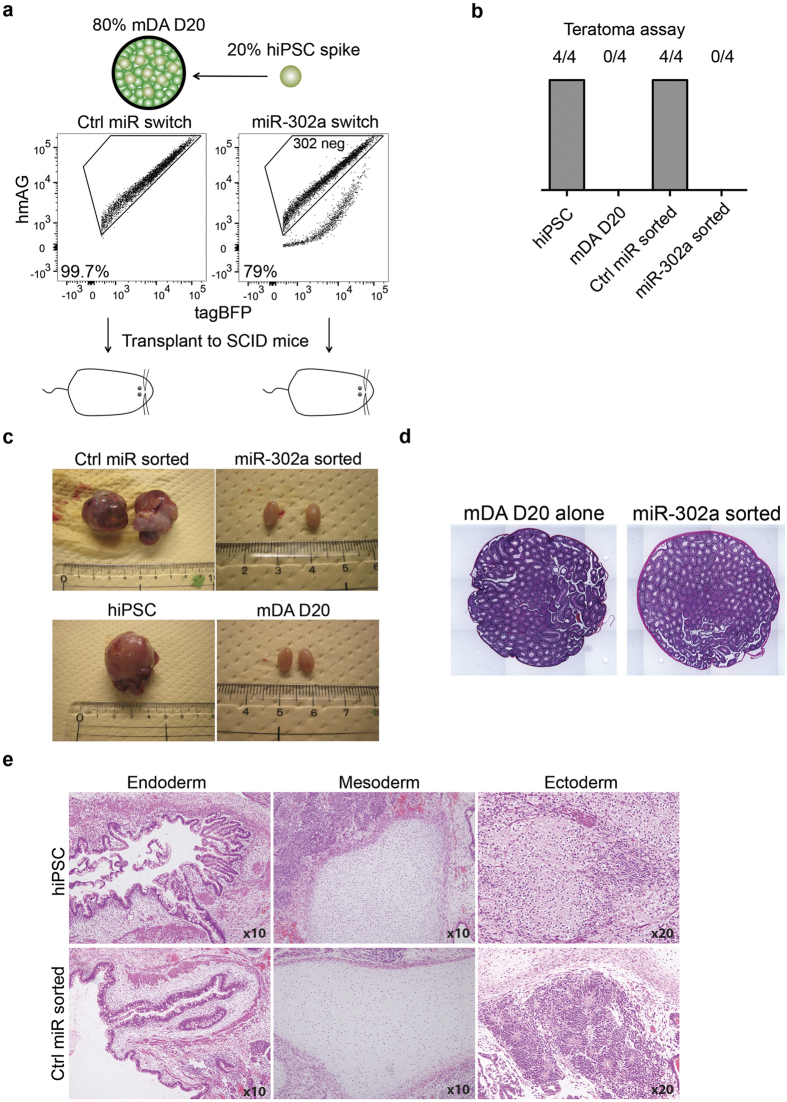
Transplantation of miR-302a switch-sorted cells. (**a**) Schematic of the experiment and representative dot plots of the sorted cells including gating. (**b**) Number of teratomas formed in mice transplanted with hiPSCs alone (positive control), mDA day 20 cells alone (negative control), Ctrl-miR switch-sorted cells, and miR-302a switch-sorted cells (n = 4 for all groups). (**c**) Photos and macroscopic inspection of the testes taken from SCID mice transplanted with sorted or unsorted cells. (**d**) Representative H&E stained cryo-sections illustrate normal testes morphology of SCID mice transplanted with mDA D20 cells alone or spiked mDA D20 cells sorted with miR-302a switch. (**e**) Representative H&E stained cryo-sections showing the generation of all three germ layers within the biopsied teratomas.

To investigate whether miR-302a switch could prevent teratoma formation in iPSC-mixed cell populations, we spiked mDA day 20 cultures with hiPSCs and then transfected the cells with either Ctrl-miRNA switch or miR-302a switch. To show that we could effectively remove the spiked hiPSC cells, we transplanted the sorted miR-302-neg cells into the testes of SCID mice for allogeneic engraftment. If any residual hiPSCs remained after sorting, these cells would form teratomas. Teratoma formation containing all cells of the three germ layers is the main standard to prove an iPSC line is truly pluripotent^22^,^23^,^24^. We chose an initial seeding density in 12-well plates of 60,000 spiked hiPSC cells with 240,000 201B7-dervied mDA cells (20% hiPSC) per well to show the strategy can handle a relatively high number of spiked hiPSCs (Fig. 5a). The sorted cells were transplanted into the testes of the animals and left to engraft for 3 months. miR-302a switch sorted cells (302-neg) effectively removed spiked hiPSCs as no teratomas were observed in all of the tested animals (Fig. 5b,c). Also, the shape and histology of the testes were normal (Fig. 5d). In contrast, all animals transplanted with either Ctrl-miR switch sorted cells or with hiPS cells only developed enlarged teratoma formation. Further microscopic observation by Haematoxylin and Eosin (H&E) staining showed these teratomas contained tissues reminiscent of the three main developmental lineages: ectoderm, mesoderm and endoderm (Fig. 5e).

### Automated elimination of residual undifferentiated cells without cell sorting

**Figure 6:**
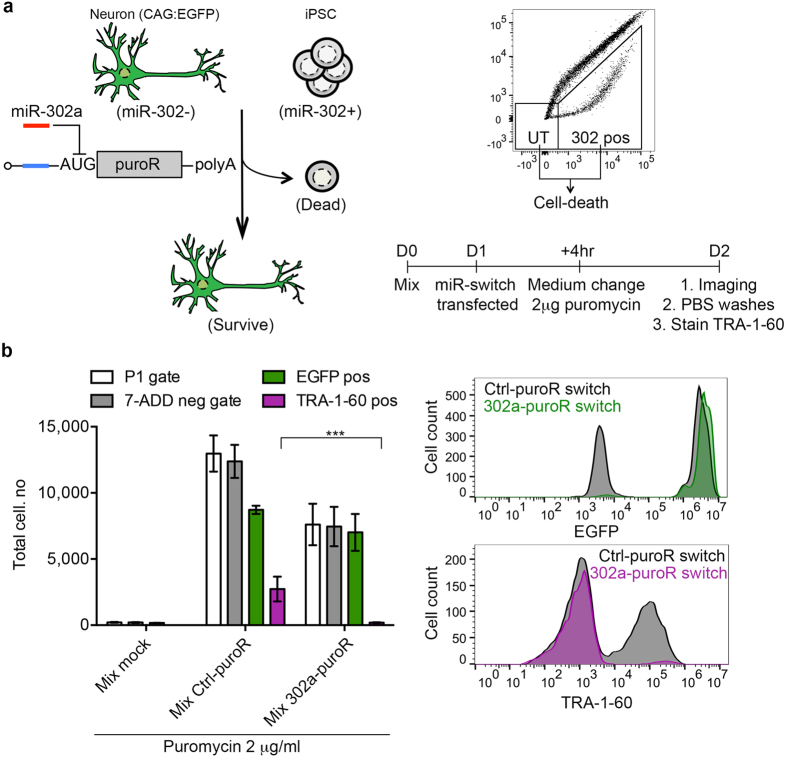
Automated purification of differentiated cells without cell sorting. (**a**) Schematic and time course of the puromycin-purification experiment. In the representative dot plot both untransfected (UT) and 302-pos populations can be removed by the addition of puromycin as they do not express the puroR gene. (**b**) LEFT Bar chart shows the total cell number from the entire cell suspension volume counted in P1/whole-cell- (white), 7-ADD neg- (grey), EGFP pos- (green) and TRA-1-60 pos- (purple) gates after the stated treatments. UPPER RIGHT Representative histograms of EGFP fluorescence (7-ADD neg-gated cells) under conditions 2 μg of puromycin with either 50 ng Ctrl-puroR switch (black shade) or 302a-puroR switch (green shade) (n = 3 for all groups). LOWER RIGHT Representative histograms of TRA-1-60 staining (7-ADD-neg gated cells) under conditions 2 μg of puromycin with either Ctrl-puroR switch (black shade) or 302a-puroR switch (purple shade). Error bars represent the SEM of three independent experiments.

A major advantage of our strategy is that we can place any gene as the reporter on the miR-302a switch. Therefore, we hypothesised that placing a simple puromycin selection circuit using puromycin-resistant mRNA (puroR) under the translational regulation of miR-302a switch would allow for automated elimination of residual hiPSCs without the need for cell sorting (Fig. 6a). We chose puromycin selection because of the ease of the design and puromycin is already used globally to form stable clones and has very little bystander effects on neighbouring cells. Firstly this design allows us to remove untransfected cells by adding puromycin. In cells that express active miR-302a, the miR-switch will repress the translation of the downstream puroR gene. Thus, upon supplementing the medium with puromycin, these cells will die. In contrast cells unresponsive to miR-302a switch will survive (Fig. 6a). Initially, we optimised the experimental conditions to assess toxicity of the puromycin. We found 2 μg/ml was sufficient to give complete toxicity for both hiPSCs and differentiated mDA cells (Supplementary Fig. 5). Approximately 10 ng of puroR mRNA (in 96-well plate scale) was enough to restore cell viability under 2 μg/mL puromycin. When regulating the puroR translation with miR-302a switch, we observed significant cell toxicity in hiPSCs but not in differentiated mDA neuronal cultures (Supplementary Fig. 5b).

Having succeeded in showing cell-specific toxicity with miR-302a-puroR switch in single culture, we then aimed to eliminate hiPSCs co-cultured with differentiated mDA cells. To eliminate the possibility that the culturing condition itself could lead to the loss of hiPSCs irrespective of the puromycin selection, we co-cultured in hiPSC culture medium rather than mDA medium or a mixture of the two. To identify the original input cells more easily, we differentiated from a 201B7 line containing a single integrated EGFP gene driven by a constitutive CAG promoter (201B7:EGFP) and stained with TRA-1–60 to identify hiPSCs (gating of populations shown in Supplementary Fig. 6). Compared to Ctrl-puroR switch-transfected co-cultures, 302a-puroR switch could efficiently eliminate TRA-1-60-pos cells to enrich EGFP-pos cells (201B7:EGFP-derived mDA cells), suggesting that it can remove undifferentiated cells without the requirement of sorting (Fig. 6b and Supplementary Fig. 6).

## Discussion

To provide effective cell therapies that use iPSC technology, the transplanted cells must be devoid of residual undifferentiated and/or partially differentiated cells that will lead to teratoma formation or graft overgrowth respectively. Making use of miRNA switches, which can be used to identify live cells based on the cells’ unique miRNA activity signatures, we set out to develop a method that is highly specific and sensitive for eliminating out pluripotent cells and differentiation-retardant cells. To our knowledge, the miR-302a switch is the only methodology that identifies, purifies, and/or eliminates target pluripotent stem cells based on intracellular signatures. The miR-302/367 cluster expression is necessary for maintaining the self-renewal of pluripotent cells and also an upstream regulator for many classical stem cell markers such as TRA-1-60, placental-like alkaline phosphatase and the intracellular transcription factors OCT3/4 and NANOG, which can only be measured in fixed or lysed cells^25^,^26^. Using this cluster, we engineered a very sensitive method that can track the dynamics of differentiating live cells. It is also noteworthy that our miR-302 switch system eliminates undesired cells based on positive selection of fluorescent-reporter mRNAs (*i.e.*, we can isolate fluorescent-reporter-positive (miR302-negative) cells). Thus, we can easily exclude both untransfected cells and miR-302-positive/undifferentiated cells.

Previous studies have reported methods to detect and/or eliminate residual hPSCs from target cell populations, including antibody staining combined with FACS^27^,^28^, small molecule- or recombinant protein-mediated hPSC staining or killing^29^,^30^,^31^,^32^,^33^,^34^, and RT-qPCR analysis of the pluripotent intracellular marker LIN28^35^,^36^. However, it is still difficult to detect undifferentiated cells (both hPSCs and partially differentiated cells) efficiently, selectively, and safely. For example, current issues surrounding the use of antibody staining, such as lot-to-lot variation^37^,^38^, non-specific binding^38^,^39^,^40^, and the retention of the antibodies on cell surface leading to localized immunogenicity^41^,^42^,^43^, further emphasises the need to develop alternative technologies. We believe our miR-switch method provides several advantages over the existing technologies as follows. (1) Using miR-puroR switch, we can avoid the necessity for cell sorting, which can be time consuming and damaging to the cells (Fig. 6). Using additional synthetic biology tools we may further tune our synthetic cell-killing circuit and more finely control the threshold between cell survival and cell death^44^. (2) Our miR-302a switch is highly sensitive and selective for the detection of hiPSCs and can be applied to any differentiation protocol because miR-302a activity can be used as a unique and robust hiPSC signature (ref. 45 and data in Fig. 1b and Supplementary Fig. 1a). In addition, we can utilize multiple miRNA signatures by introducing multiple miRNA switches into the cells if needed^12^,^13^. (3) Our method has a high resolution for identifying cell sub-populations. Therefore the miR-302a switch may be used for both the detection of purely spiked hiPSCs^27^,^28^,^29^,^30^,^31^,^32^,^33^ and partially differentiated cells or cells that slowly differentiate. Thus, our switch may be used to investigate the dynamics of differentiation and selection of optimal clones for directed-differentiation by monitoring miR-302a activity. For example, we consistently observed miR-302a switch-responsive cells up to day 9 of the mDA protocol despite being TRA-1-60 negative (Fig. 2d,e). We also observed that the 1231A3 hiPSC consistently gives higher mDA differentiation efficiency compared to 201B7 hiPSCs. If comparing miR-302a-responsive cells over time, the 1231A3 hiPSCs also differentiate quicker than 201B7 hiPSCs. We believe removing residual miR-302a-responsive cells at earlier time points (*e.g.*, day 5 in Fig. 2d) and re-culturing the negative fraction (miR-302a non-responsive cells) may lead to greater differentiation efficiencies. (4) Standard laboratory equipment and widely available kits are all that is required for our method. The amount of transfected RNA required is also minimal (500 ng of switch RNA/1 × 10^6^ cells), therefore providing competitive cost effectiveness. We consistently observed transfection efficiencies of approximately 95% with multiple hiPSC lines and their derived cell types (Supplementary Table 3). Since antibody staining is not 100% efficient also, we believe our transfection efficiencies are an acceptable standard seeing that we can easily kill untransfected cells using puromycin selection. (5) Our miR switches should be safe to the cells because they are encoded on modRNA, which circumvents the issue of possible genomic integration seen with DNA encoded regulatory circuits^44^,^46^. Transfection of modRNA is a widely used method and several studies have shown it has very little if any toxicity on mammalian cells^46^. The transient expression of our miR switches also serves as an advantage, as we only need isolate the cells in a brief window of time. In fact, we previously have shown that miR switches do not significantly alter the steady-state expression of the target miRNA or expression of their target mRNA^12^.

The miRNA-302/367 cluster is so directly coupled with the stem-cell identity to the extent it can greatly increase reprogramming efficiency^14^,^15^,^17^,^47^,^48^, and so our miR-302a switch may also be used for purposes other than removing undifferentiated cells. Using an imaging cytometry approach^13^, we propose the possibility of identifying newly reprogrammed hiPSC cells from somatic cells *in situ*. Current methods require extensive training, as they require subjectively assessing the morphology of the cells/colonies by eye and then later confirming through replica plating and fixed-cell ALP staining^21^,^49^,^50^. Tracking the miR switch response of differentiated cells over time will also reveal information on the dynamics and assess different protocols for directed differentiation. Finally, our technology may provide greater insights into miRNA activity and pluripotency (*e.g.*, the primed and naïve states of cells)^51^,^52^,^53^,^54^,^55^. So far, microarray and RNA-seq technologies have revealed miRNA expression differences between primed and naïve states of pluripotency in both mouse and humans, in particular members of the miR-302 and miR-371 (miR-290 in mouse) clusters^54^,^56^,^57^. However it is important to verify whether the expression differences translate to true activity differences, which we can do using our miR switch technology. A recent report has found approximately 60% of a cell’s detectable miRNAs have no or relatively weak activity^58^. Thus, our miR switch can be used as a basic research tool to investigate the dynamics of differentiation and reprograming of live cells using intracellular information.

## Methods

### Cell culture and reagents

Human iPSC lines (201B7, 1231A3, and 1383D7) were a kind gift from Dr. Masato Nakagawa & the CiRA FiT facility (Kyoto University). Normal human dermal fibroblast cells (NHDF; CC-2511) were purchased from Lonza (USA), and HeLa CCL-2 cells were obtained from the ATTC depository. StemFit AK03 medium was purchased from Ajinomoto Pharmaceuticals (Japan). Hank’s balanced salt solution (HBSS), Glasgow’s modified essential medium (GMEM), KnockOut serum replacement (KSR), Neurobasal A medium, and NB27 neural supplement were purchased from Life Technologies (USA). 100 sodium pyruvate solution, 1000×β-mercaptoethanol solution, and 100X β-glutamine solution were purchased from Sigma Aldrich (USA). iMatrix-511-E8 fragment was purchased from Nippi (Japan). Other tissue culture reagents were purchased from Life Technologies unless stated otherwise. Recombinant human FGF8 (Cat#063-04371), Purmorphamine (Cat#166-23991), and Y-27632 (Cat#08945-84) were purchased from Wako Pharmaceuticals (Japan). Dibutyl-cyclicAMP (Cat#D0627-250MG) and ascorbic acid salt (Cat#A4034-100G) were purchased from Sigma. LDN193189 (Cat#04-0074), A83-01 (Cat#04-0014), and CHIR99021 (Cat#04-0004-10) were purchased from Stemgent. Recombinant human BDNF (Cat#248-BD-025) and recombinant human GDNF (Cat#212-GD) were purchased from R&D (USA). mirVana miRNA Mimic, Negative Control #1, mirVana miRNA Inhibitor Negative Control, hsa-miR-302-5p mimic (Pre-miR/Anit-miR ID:MC12557), and 302a-5p inhibitor (Pre-miR/Anti-miR ID:MH12557) were purchased from Applied Biosystems and used at the stated picomole amount.

### hiPSC maintenance and differentiation

Feeder-free (Ff) hiPSC lines were grown attached to plates coated with laminin-511-E8 fragment (iMatrix, Nippi) in xeno-free defined StemFit medium AK03 medium (Ajinomoto). These methods are designed to obtain clinical-grade hiPSC with standard operating protocols^59^. hiPSCs were passaged once every 8 days. 6-well plates were coated at 37 °C for at least 1 hr before passage with iMatrix-511(E8) at a density of 0.5 μg/cm^2^ diluted in sterile PBS. The cells were passaged as previously published^59^. For mDA differentiation, 6-well plates were coated the same as above with iMatrix-511-E8 and hiPSCs seeded at density of 5 × 10^6^ cells/well. The cells over 12 days were induced to dopaminergic neurons in xeno-free conditions^59^. After 12 days, the cells were passaged and maintained in Neurobasal medium supplemented B27, GDNF, BDNF, ascorbic acid and dibutyl-cyclicAMP. Transfection of these mDA cells was either carried out in the same medium or the stated medium otherwise.

For spontaneous differentiation, Ff-hiPSC cells were plated onto 6-well coated plates at a density of 8 × 10^4^ cells/well and maintained in StemFit AK03 medium without the addition of bFGF for 14 days. After 7 days, cells were passaged using Accumuax to a new 6-well coated plate at density of 2.5 × 10^5^ cells/well.

The 201B7-DD″ line was classified as differentiation-defective because this line consistently contains ~10% TRA-1-60-pos cells after differentiation. In contrast its parental line, 201B7, has less than 1% TRA-1-60-pos cells after differentiation. The 201B7-DD″ line was established by FACS-sorting for TRA-1-60-pos cells after each round of spontaneous differentiation (Supplementary Fig. 2). After the first round of differentiation, TRA-1-60-pos cells (approximately 8%) were seeded and grown under normal hiPSC culture conditions and passaged once. The passage two cells were taken through a second round of differentiation. TRA-1-60-pos cells (approximately 14%) were seeded and grown under normal hiPSC culture conditions and passaged once to establish 201B7-DD″ before differentiating again for miR switch experiments.

### Assessing differentiation efficiency

During each passage at D12 and D20, the efficiency of differentiation was assessed by staining the bulk cells with a CORIN antibody from R&D Systems (MAB2209), a floor-plate marker and a PSA-NCAM antibody, which marks the neural lineage. Approximately 5 × 10^5^ cells in 200 μL of stain buffer (with ROCK inhibitor) in parallel were stained with either 1/200 of mouse anti-human CORIN antibody (R&D Systems) or 1/100 of mouse monoclonal anti-human PSA-NCAM (MAB5324, Millipore). Alexa 488-conjugated anti-rat IgG or anti-mouse IgM antibodies were used at 1/400 for CORIN and PSA-NCAM, respectively. Cells were stained for 30 min at 4 °C. Cells were washed twice in HBSS medium. Dead cells were stained with 7-AAD for 10 min prior to FACS analysis.

Cells were analysed on either a BD FACS aria-II or BD Accuri with standard filters for FITC and 7-AAD signals. Gates were set up to eliminate dead cells based on SSC-A intensity (P1) and to remove doublets from FSC-W vs FSC-H (P2) and SSC-W vs SSC-H (P3). Finally 7-AAD high cells were excluded leaving the ‘LIVE’-cell gate (P4/LIVE). 20,000 P4-gated cells were analysed.

### Template generation and *in vitro* transcription (IVT)

In this study we *in vitro* transcribed modRNA and then transfected to the cells. The general method for the generation of the modRNA was detailed previously^12^,^13^. Three separate PCR reactions were carried out to produce a designed 5′-untranslated region, ORF and a designed 3′-UTR PCR products (Supplementary Table 1). The 5′-UTR products were amplified from the oligos listed using either TAP_T73GC (Ctrl-miR switch) or GCT7pro_5UTR2 (302/367-miR switch) forward primer and Rev5UTR reverse primer. Similarly, the 3′-UTR products were amplified from the oligos listed using the Fwd3UTR forward primer and 3′UTR_2T20Trev reverse primer. The ORF products were amplified using the forward and primers listed. The following products were then combined into one full-length PCR product using fusion PCR with an additional 120 nt polyA sequence, which extends the stability of the transcribed mRNA. The 5′ end contains a T7 promoter, which can bind to the T7 RNA polymerase used in commercial IVT kits. For the miR-responsive switch, anti-sense sequence for the target miRNA was inserted into an optimised position in the 5-UTR to allow for translational repression of the downstream reporter if bound to this miRNA. The target sites for hsa-miR-302a-5p and 367-3p are underlined and in bold in the primer list (Supplementary Table 1).

### RT-qPCR

Relative miRNA expression was carried out using TaqMan small RNA assays for hsa-miR-302a-5p (Assay ID: 002381), 367-3p (Assay ID: 000555), and RNU6B (Assay ID: 001093). Cells for RNA analysis were first pelleted, medium aspirated, and then snap frozen in liquid nitrogen and stored at −80 °C. Pellets were thawed on ice and then rapidly resuspended in Trizol for RNA extraction following the manufacturer’s protocol. Pelleted RNA was resuspended in RNAse-free water treated with TURBO DNase inactivation kit following the manufacturer’s protocol. Total RNA was assessed by Agilent Bioanalyzer with RIN >8 for all samples tested. First strand synthesis was carried out using 10 ng of RNA, the miRNA-specific TaqMan RT primer and the MultiScribe RTase kit. PCR amplification was done by miRNA-specific TaqMan fluorescent probes and TaqMan Fast Universal PCR Master Mix (2X), no AmpErase UNG on Applied Biosystems StepOne in realtime. miRNA was normalized to the small RNA, RNU6B.

For messenger RNA analysis, reverse transcription was carried out using 1 μg of RNA, random hexamer primers and the TOYOBO ReverTra Ace-α kit following the manufacturer’s instructions. Primers for real-time amplification are listed in Supplementary Table 2 and were found to give efficiencies close to 2 from initial optimisation. 10 ng of cDNA input was amplified with Power SYBR Green PCR Master Mix for 40 cycles followed by melt-curve analysis. Target mRNA was normalized to GADPH. Raw Ct values were analysed and presented using the Pfaffle ΔΔCt method^60^.

### RNA Transfection

A day before transfection, cells were passaged as detailed above and seeded onto iMatrix-511 (E8) coated 24-well plates at density from 1–2 × 10^5^ cells/well in differentiation medium (with ROCK inhibitor). The following day, without changing the medium, modRNA was transfected to the cells using StemFect reagent (Stemgent) following the online protocol. Briefly for each transfection, in one tube (tube A), 1 μL of reagent was mixed with 12.5 μL of StemFect buffer and incubated for 5 min at room temperature (RT). In another tube (tube B), up to 400 ng of modRNA was mixed with 12.5 μL of StemFect buffer. Tube B was added to tube A in a drop-wise manner, and the mixture was briefly flick-mixed and incubated for 10 min at RT. The transfection complex was added to the cells in a drop-wise manner and the plate was agitated before being placed into the incubator. Four hours later, the old medium containing the complex was aspirated and fresh differentiation medium (without ROCK inhibitor) was added. For the experiments in Fig. 4, transfection complexes were formed as above but were added to the cell suspensions during seeding of the cells to culture plates on the same day of passaging.

Cells were analysed the following day by FACS analysis on either a BD FACS Aria-II or BD Accuri using standard filter sets and similar gating for antibody staining. The transfection efficiency was judged against mock-transfected cells. In the case of reverse transfection, the complex was mixed with the cell suspension on the day of passage when seeded to new plates.

To control for variation in the transfection efficiency between cells, signals from the switch were normalized to that of an internal control. For the tagBFP-pos population (usually >90% of live-cells), the geometric mean of the fluorescence intensity (arbitrary units) for hmAG was divided by that of tagBFP to give the normalized translation efficiency. The normalized translation efficiency was then expressed as percentage of Ctrl-miR switch transfected cells in the bar charts.

### *In vivo* tumorigenicity assay

Purified or non-purified cells were injected into the testes of SCID mice. Cells were checked for mycoplasma presence before transplanting in order to prevent infection. 201B7 hiPSCs were differentiated to mDA for 20 days. The differentiated cells were passaged and seeded into 12-well plates, some of which were spiked with 201B7 hiPSCs, at a final density of 0.75 × 10^5^ cells/cm^2^. The following day, wells spiked with hiPSC were transfected with either 180 ng of Ctrl-hmAG1 or 302a-5p-hmAG1 and 400 ng of tagBFP. After 24 hr, cells were sorted using FACS Aria-IIu and collected into StemFit AK03 medium containing ROCKi. 2 × 10^4^ 201B7 hiPSCs were injected into both testes of 3 animals. 2 × 10^5^ of non-sorted mDA D20 cells, Ctrl-miR switch-sorted hiPSC-spiked mDA culture, and miR-302a switch-sorted hiPSC-spiked mDA culture were injected into both testes of 4 animals for each group. After 3 months, animals were sacrificed, and the testes dissected out from the body. Images were taken to record macroscopic appearance and then the tissue was fixed in saline solution, sectioned and H&E stained to observe microscopic tissue morphology.

The animal experiments were performed in accordance with the guidelines and regulations for Animal Experiments of Kyoto University and approved by the same institution.

### Puromycin purification

Single and co-cultures cells were treated as shown in Fig. 6a and Supplementary Fig. 5a. One day before transfection, cells were passaged and seeded into 24-well plates. Even though we found the action of puromycin to override the anti-apoptotic effect of the ROCK inhibitor used for hiPSC passaging, we decided to change the medium 2 hr before transfection to remove the inhibitor. Four hours after transfection, the medium was changed again and included puromycin at 2 μg/mL. For the WST-1 cell viability assay (Roche), fresh medium containing WST-1 substrate was added 2 days after transfection and incubated for 4 hr before reading absorbance at the recommended wavelength on TECAN microplate reader (Infinite M1000).

For the experiments in Fig. 6, cells were washed twice with 1xPBS one day after transfection to remove dead and dying cells. Cells were harvested with trypsin, pelleted and re-suspended in stain buffer (HBSS and 2% knockout serum). Cells were then stained with an Alexa 647-conjugated anti-TRA-1-60 antibody (BD Laboratories) for 20 min at 4 °C, washed twice with stain buffer and finally stained with the 7-AAD dye in 150 μl stain buffer. The entire cell suspension volume of harvested cells was analysed on a BD Accuri cell cytometer. Representative dot plots and corresponding gating can be seen in Supplementary Fig. 6 for the following parameters: SSC-A vs. 7-ADD, and TRA-1-60 vs. EGFP. The live cell gate, P1-gate, was established on FSC vs. SSC signal to eliminate cell debris and most of the dead cells and on the SSC vs. 7-AAD signal to remove any remaining dead intact cells.

### Statistical analysis

FACS dot plots and histograms were produced in FlowJo software for Mac. Significant differences between means were statistically tested to the 5% confidence level using either non-parametric *t*-test when referring only to the control or one-way ANOVA followed by post-hoc test when comparing all groups. The levels of significance are denoted as; *P < 0.05, **P < 0.01, and ***P < 0.001. The statistical analysis is based on the means generated from at least three independent experiments performed on separate days.

## Additional Information

**How to cite this article**: Parr, C. J. C. *et al*. MicroRNA-302 switch to identify and eliminate undifferentiated human pluripotent stem cells. *Sci. Rep.* **6**, 32532; doi: 10.1038/srep32532 (2016).

## Supplementary Material

Supplementary Information
