# Supplementary material for: MicroRNA-302 switch to identify and eliminate undifferentiated human pluripotent stem cells
Source: Sci Rep. 2016 Sep 9;6:32532. doi: 10.1038/srep32532 (PMC5016789; doi:10.1038/srep32532)
Supplement: Supplementary Information [file srep32532-s1.pdf]

MicroRNA-302 switch to identify and eliminate undifferentiated human pluripotent stem cells

Callum J.C Parr[1,*], Shota Katayama[1,*], Kenji Miki[1], Yi Kuang[1], Yoshinori Yoshida[1], Asuka Morizane[2], Jun Takahashi[2], Shinya Yamanaka[1,3], and Hirohide Saito[1].

[1]Department of Life Science Frontiers, Center for iPS Cell Research and Application (CiRA), Kyoto University, Kyoto, Japan.
[2]Department of Clinical Application, Center for iPS Cell Research and Application (CiRA), Kyoto University, Kyoto, Japan.
[3]Gladstone Institute of Cardiovascular Disease, San Francisco, CA 94158, USA
[*]These authors contributed equally

*Corresponding author. All correspondence should be directed to: hirohide.saito@cira.kyoto-u.ac.jp

Supplementary Figures 1-6

Supplementary Table 1-3

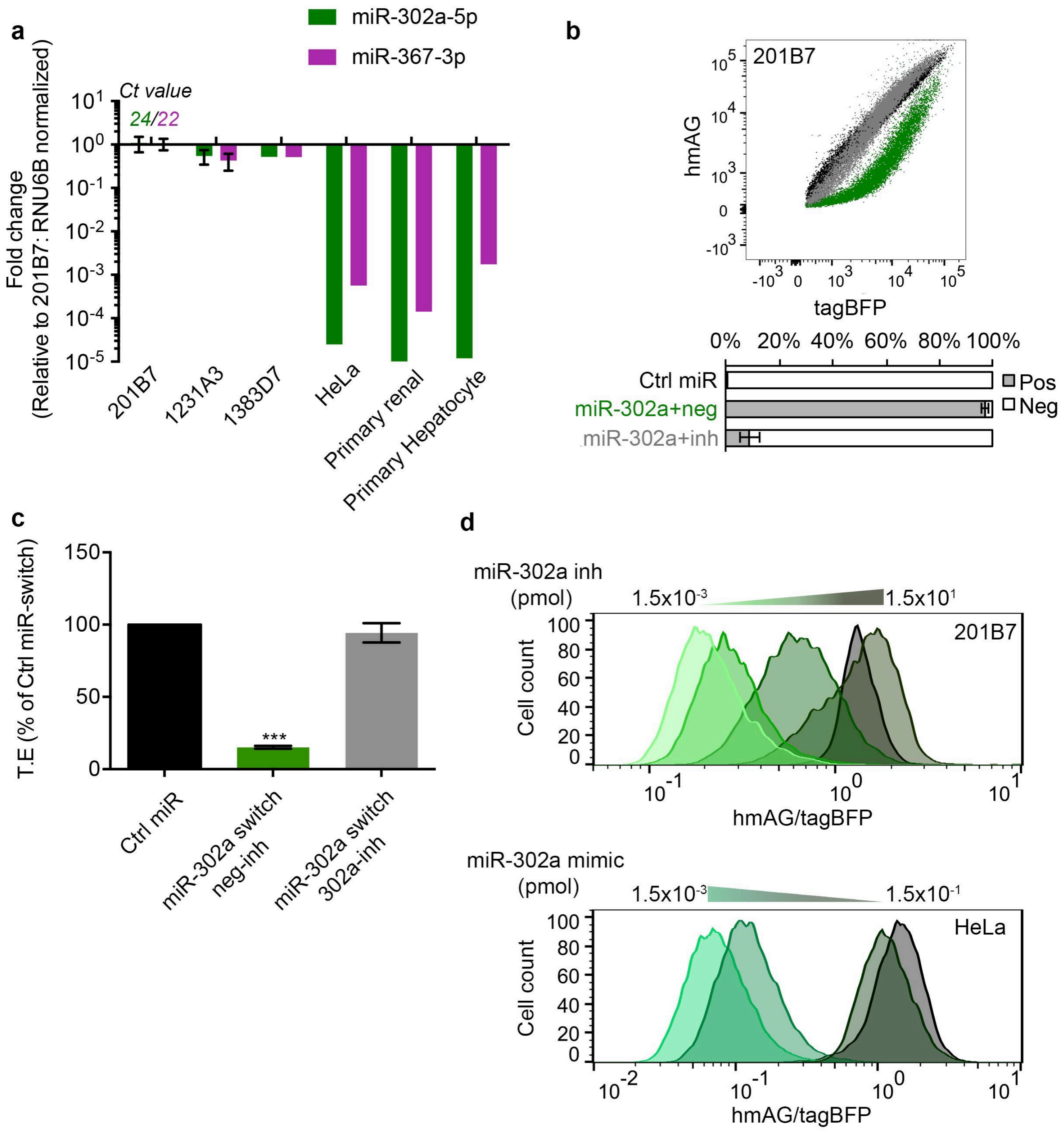

Supplementary Figure 1: Specificity of miR-302/367 switches used in Fig. 1

(a) hsa-miR 302a-5p and 367-3p expression relative to 201B7 cells from two other Ff-hiPSC lines, 1231A3 and 1383D7, HeLa, and primary renal and hepatocyte cells. (b) Representative dot-plot of 201B7 cells transfected with either Ctrl-miR switch (black), miR-302a switch and 15 pmol of control miRNA inhibitor (green) or miR-302a switch and 15 pmol of miR-302a inhibitor (grey). Below, bar chart shows percentage fraction of 302-pos and 302-neg cells (n=3 for all groups). (c) Percentage of Ctrl-miR switch translation efficiency 201B7 cells transfected with either Ctrl-miR switch (black), miR-302a switch and 15 pmol of control miRNA inhibitor (green) or miR-302a switch and 15 pmol of miR-302a inhibitor (grey). (d) *UPPER representative* hmAG1/tagBFP histogram of 201B7 cells co-transfected with either the miR-302a switch with 15 pmol control miRNA inhibitor (black shaded) or miR-302a switch and the miR-302a inhibitor at $1.5x10^{-3}$ to $1.5x10^{1}$ pmol (shades of green). *LOWER* representative hmAG1/tagBFP histograms and dot-plots of HeLa cells transfected with miR-302a switch with 1.5 pmol control miRNA inhibitor (black shade) and miR-302a switch and the miR-302a mimic at $1.5x10^{-3}$ to $1.5x10^{-1}$ pmol (shades of green). Error bars represent the SEM of three independent experiments.

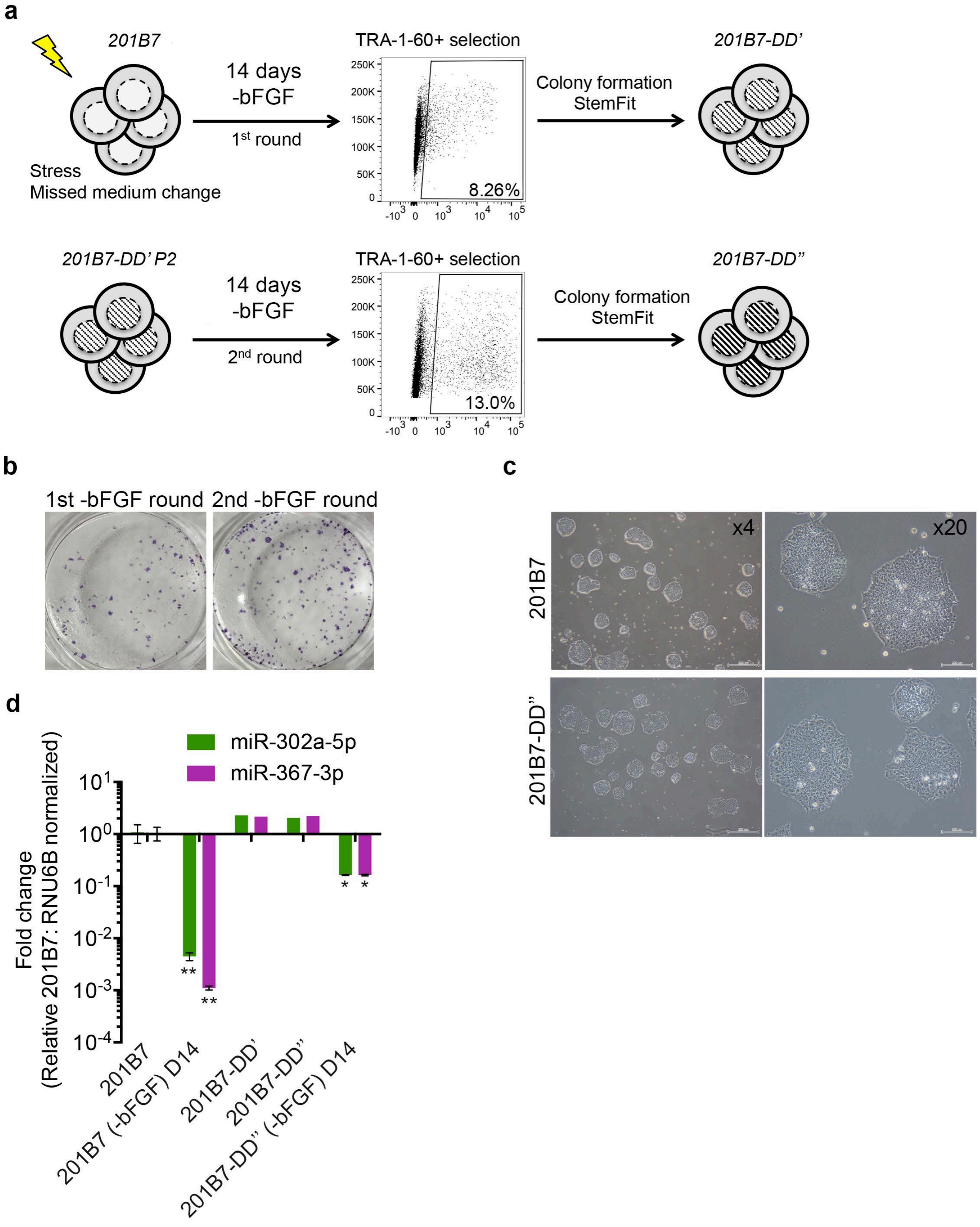

Supplementary Figure 2: Generation of 201B7 differentiation-defective'' line

(a) Scheme of 201B7 differentiation-defective'' (201B7-DD'') generation. TRA-1-60 positive selection after two differentiation rounds in hiPSC medium minus –bFGF for 14 days selected for differentiation-defective (DD'') cells. (b) ALP^+ colony staining after each differentiation round. (c) Morphology of 201B7 and 201B7-DD'' cells at x4 and x20 magnification. (d) Relative miR-302a-5p and 367-3p expression for 201B7, spontaneously differentiated 201B7 cells, 201B7-DD', 201B7-DD'', and spontaneously differentiated 201B7-DD''. Repeated once for 201B7-DD' and -DD''. Error bars represent the SEM of three independent experiments.

a

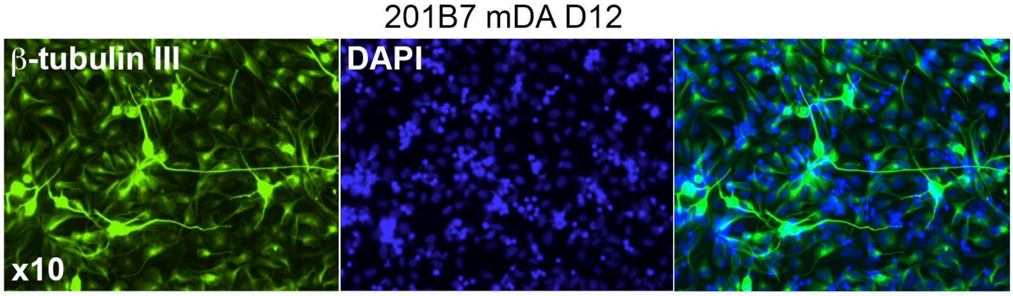

b

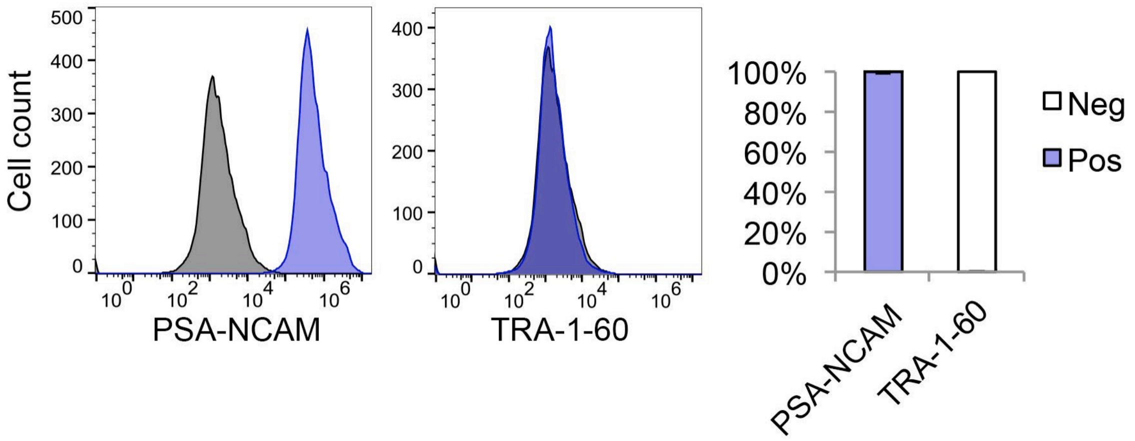

Supplementary Figure 3: Antibody staining of 201B7-derived mDA D12 cells

(a) Representative fluorescent images of 201B7-derived mDA cells stained with a β-tubulin III antibody at day 12. (b) Representative histograms of PSA-NCAM and TRA-1-60 staining (IgG control shown in black shade, positive stain in blue shade) and to the right percentage fraction of positively stained cells (n=3).

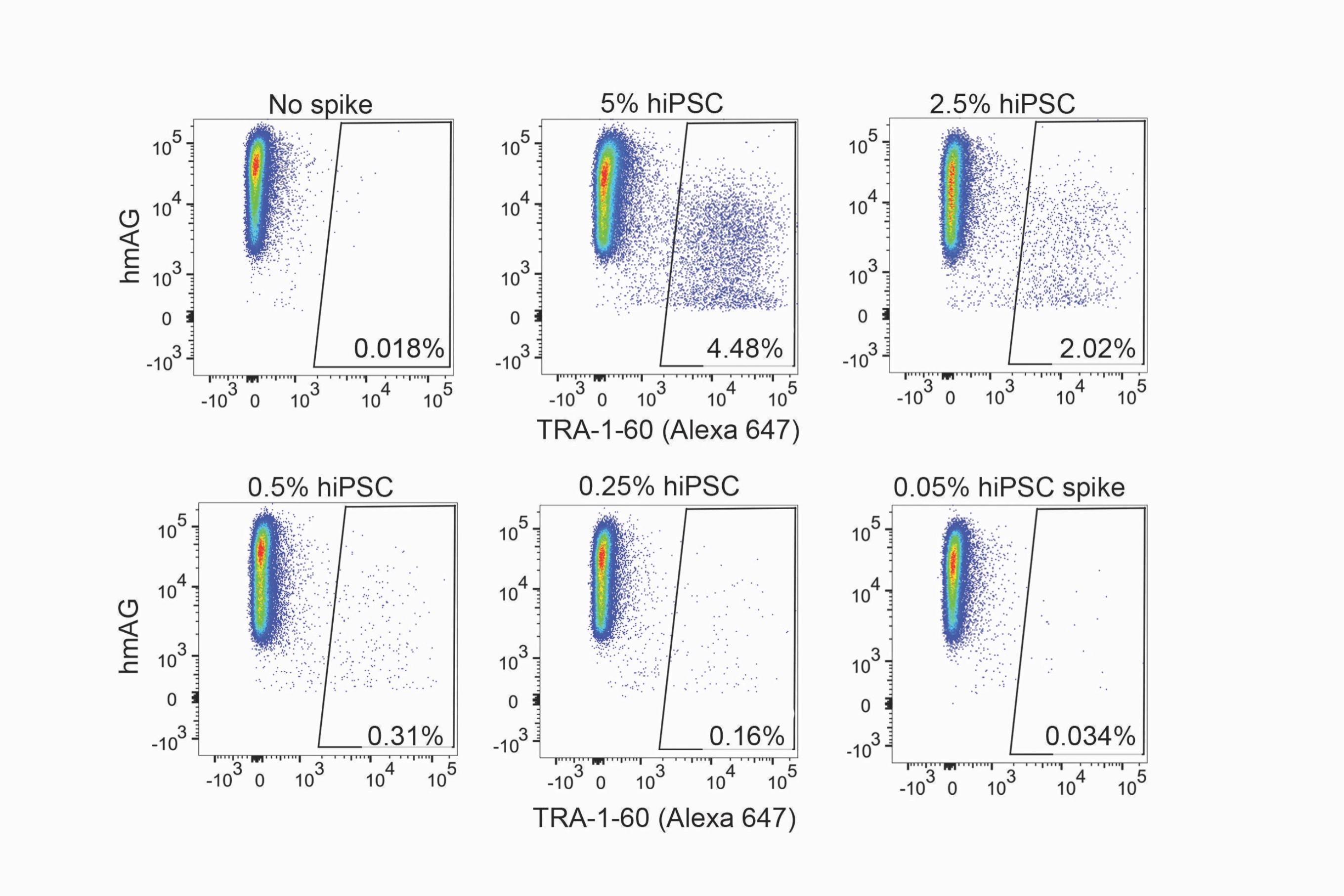

Supplementary Figure 4: Representative TRA-1-60 staining of hiPSC-spike experiment shown in Fig. 3

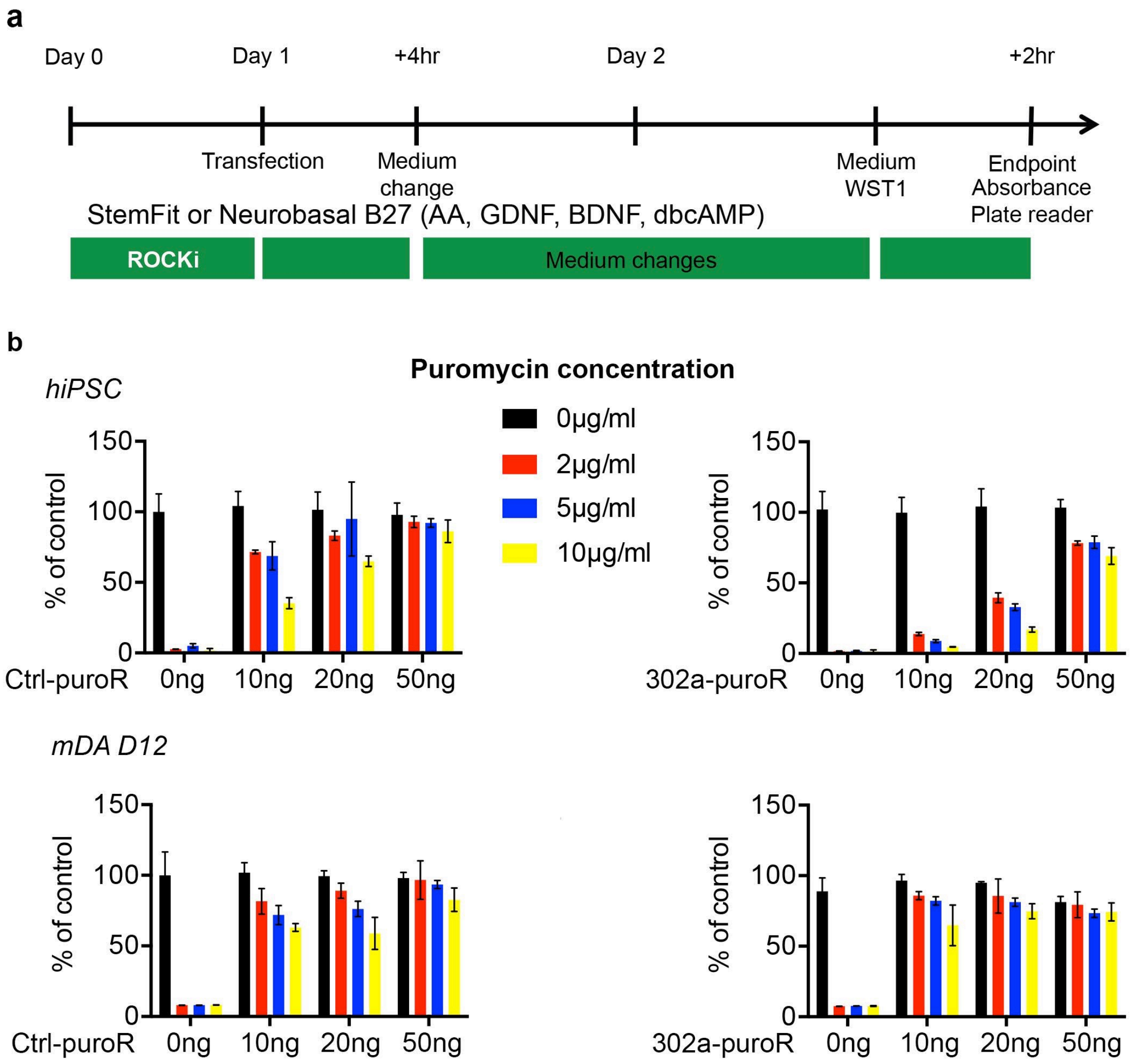

Supplementary Figure 5: Optimization of puromycin concentration and puroR amount
(a) Scheme of treatment for cells for puromycin and puroR mRNA optimization in hiPSC and 201B7-derived mDA day 12 cells. (b) Cell viability as measured by WST-1 assay and expressed as percentage of control (mock transfected, 0 μg/mL puromycin).

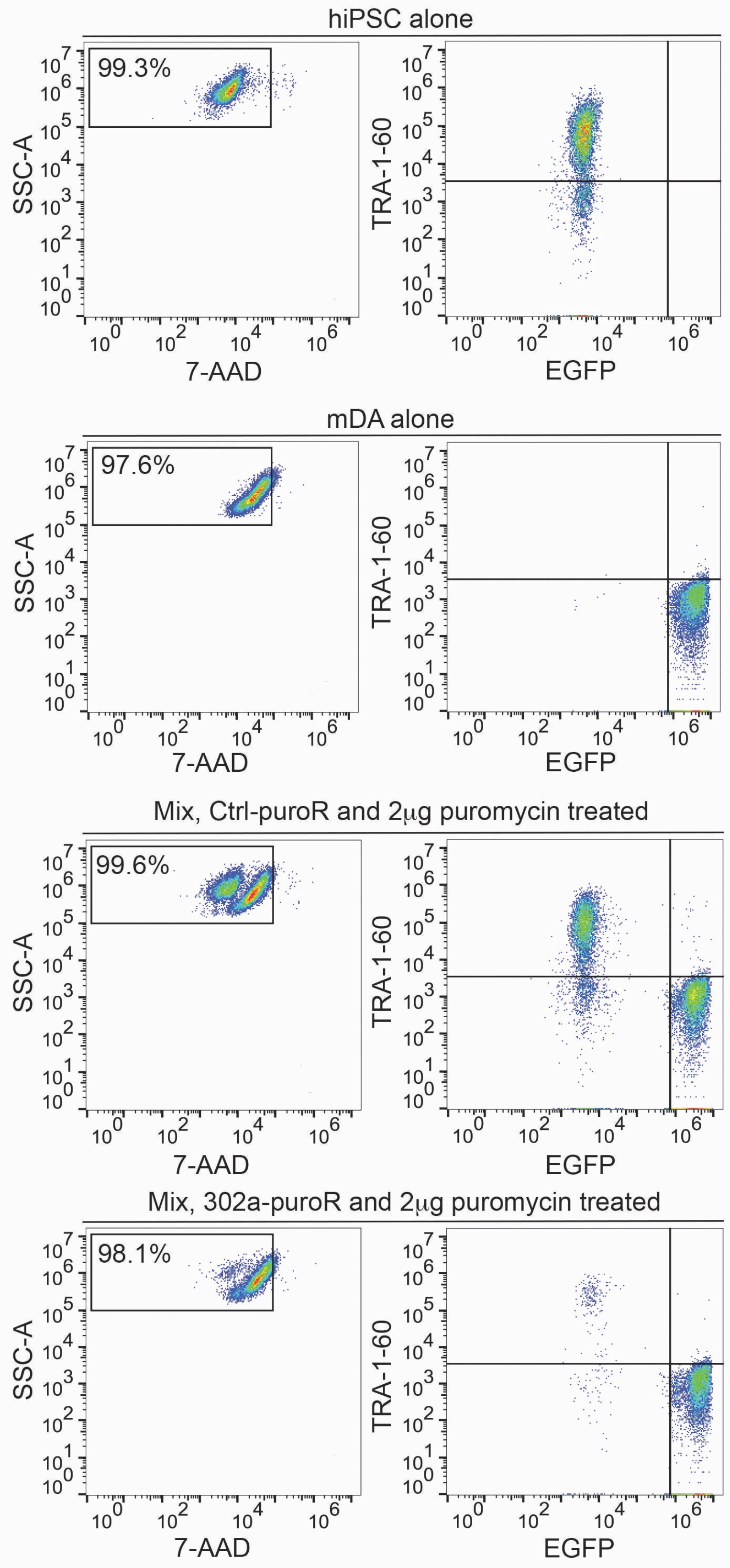

Supplementary Figure 6: Gates used in flow-cytometer analysis shown in Fig. 6
Representative dot-plots and gating. 7-AAD gate excludes dead-cells. Quadrant gates set for TRA-1-60 positive and EGFP positive cells.

Oligo		
Name	*Type*	*Sequence (5'>3')*
IVT_5prime_UTR	5'UTR	CAGTGAATTGTAATACGACTCACTATAGGGCGAATTAAGAGAGAAAAGAAGAGTAAGAAGAAATATAAGACACCGGTCGCCACCATG
5UTRtemp_T302a-5p	5'UTR	CGACTCACTATAGGTTCCGCGATCGCGGATCCAGCA**AGTACATCCACGTTTAAGT**AGATCCACCGGTCGCCACCATG
5UTRtemp_T367-3p	5'UTR	CGACTCACTATAGGTTCCGCGATCGCGGATCC**TCACCATTGCTAAAGTGCAATT**AGATCACACCGGTCGCCACCATG
IVT_3prime_UTR	3'UTR	TCTAGACCTTCTGCGGGGCTTGCCTTCTGGCCATGCCCTTCTTCTCTCCCTTGCACCTGTACCTCTTGGTCTTTGAATAAAGCCTGAGTAGG
**5'UTR, 3'UTR generation**		
Name	*Type*	*Sequence (5'>3')*
TAP_T73GC	5'UTR	CAGTGAATTGTAATACGACTCACTATAGGGC
GCT7pro_5UTR2	5'UTR	GCTAATACGACTCACTATAGGTTCCTTAATCGCGGATCC
Rev5UTR	5'UTR	CATGGTGGCGACCGGTGTCTTATATTTCTTCTTACTC
Fwd3UTR	3'UTR	TCTAGACCTTCTGCGGGGC
3'UTR_2T20T_rev	3'UTR	TTTTTTTTTTTTTTTTTTTTCCTACTCAGGCTTTATTCAAAGACCAAG
3UTR120A	3'UTR	TTTTTTTTTTTTTTTTTTTTTTTTTTTTTTTTTTTTTTTTTTTTTTTTTTTTTTTTTTTTTTTTTTTTTTTTTTTTTTTTTTTTTTTTTTTTTTTTTTTTTTTTTTTTTTTTTTTTTTCCTACTCAGGCTTTATTCA
**ORF amplification (Fwd and Rev primer)**		
Name	*ORF*	*Sequence (5'>3')*
hmAG1_IVTfwd	hmAG1	CACCGGTCGCCACCATGGTGAGCGTGATCAAGCCCG
hmAG1_IVTrev	hmAG1	GCCCCGCAGAAGGTCTAGATTCACTTGGCCTGGCTGGGC
tagBFP_fwd	tagBFP	CACCGGTCGCCACCATGGGATCCAGCGAG
TAP_IVT_rev	tagBFP	GCCCCGCAGAAGGTCTAGAtcactcgagatgcatatgagatc
ORF_puroR_fwd	PuromycinR	CACCGGTCGCCACCATGaccgagtacaagcccacg
ORF_puroR_rev	PuromycinR	CACCGGTCGCCACCATGaccgagtacaagcccacg

Supplementary Table 1: Primers and oligos used for switch generation
Sequences underlined and in bold are antisense sequences to target miRNA.

Name	Sequence (5'--->3')	GC (%)	Tm °C	length	Amplicon (bp)
GAPDH Fw Ex2-3	GGTCGGAGTCAACGGATTTG	55	58	20	174
GAPDH Rv Ex4	TCAGCCTTGACGGTGCCATG	60	60	20	174
OCT4 Fw	AGACCATCTGCCGCTTTGAG	55	58	20	66
OCT4 Rv	GCAAGGGCCGCAGCTT	69	58	16	66
NANOG Fw	GGCTCTGTTTTGCTATATCCCCTAA	44	59	25	82
NANOG Rv	CATTACGATGCAGCAAATACGAGA	42	57	24	82
LIN28A Fw	CGGGCATCTGTAAGTGGTTC	55	57	20	191
LIN28A Rv	CAGACCCTTGGCTGACTTCT	55	58	20	191
Sox1 Fw	GCGGAGCTCGTCGCATT	65	58	17	76
Sox1 Rv	GCGGTAACAACTACAAAAAACTTGTAA	33	56	27	76
PAX6 Fw	ACCCATTATCCAGATGTGTTTGCCCGAG	50	63	28	317
PAX6 Rv	ATGGTGAAGCTGGGCATAGGCGGCAG	62	67	26	317
EN1 Fw	TGGGTGTACTGCACACGTTATTC	48	59	23	145
En1 Rv	GGAACTCCGCCTTGAGTCTCT	57	60	21	145
NURR1 fw	CGAAACCGAAGAGCCCACAGGA	59	65	22	101
NURR1 rv	GGTCATAGCCGGGTTGGAGTCG	64	65	22	101
LMX1a Ex8 Fw	GATCCCTTCCGACAGGGTCTC	62	62	21	175
LMX1a Ex9 Rv	GGTTTCCCACTCTGGACTGC	60	60	20	175
CORIN Fw	CACAGCCAGGGTCTGGTGGAATGCAG	62	67	26	187
CORIN Rv	GAGAGCTACCACCACATGAATCAAGG	50	62	26	187
TH Fw Ex5	TCATCACCTGGTCACCAAGTT	48	57	21	125
TH Rv Ex6/7	GGTCGCCGTGCCTGTACT	67	60	18	125
MAP2 Fw	GGATCAACGGAGAGCTGAC	58	58	19	99
MAP2 Rv	TCAGGACTGCTACAGCCTCA	55	58	20	99

Supplementary Table 2: qPCR primers used in this study

	hiPSC			D14 mDA			D14 Spon. Diff	HeLa
	201B7	*1231A3*	*1383D7*	*201B7*	*1231A3*	*1383D7*		
tagBFP (%)	93.25	93.38	96.07	98.63	98.18	99.23	98.00	99.78

Supplementary Table 3: Transfection efficiency
Percentage tagBFP-pos from transfected hiPS, D14 differentiated, and HeLa cells.
